# Comparative Responses of Self‐Regulated Exercise, Thermoregulation, and Neuromuscular Function to Monophasic Oral Contraceptives Across One Menstrual Cycle

**DOI:** 10.1002/ejsc.70083

**Published:** 2025-12-20

**Authors:** Katie James, Jack Cannon, Frank E. Marino

**Affiliations:** ^1^ School of Allied Health Exercise & Sports Sciences Bathurst Australia; ^2^ School of Rural Medicine Charles Sturt University Orange Australia

**Keywords:** exercise, menstruation, oral contraceptives, thermoregulation, women

## Abstract

This study investigated the effect of monophasic oral contraceptive (OC) use on self‐paced cycling performance, thermoregulation, and skeletal muscle strength and contractile properties in healthy women. Eight physically active women performed 30 min fixed intensity cycling at 50% *W*_max_, followed by a 30 min self‐paced time trial (TT) with each section interspersed with a 30 s maximal sprint at 9, 19, and 29 min. The tests were conducted in different ambient conditions corresponding to either baseline day 1 (20°C, BASE), day 8 (20°C COOL), or day 18 (32°C WARM) of 1 month pill cycle. Core (*T*_c_) and skin (*T*_sk_) temperatures, heart rate, perceptual, neuromuscular responses, and serum cortisol (CORT) were measured multiple times throughout the trials and postexercise. Time trial performance remained unchanged across conditions (range 10.9–11.1 km) although *T*_c_ was elevated in WARM in the final 15 min of self‐paced exercise, reaching 38.5°C (*p* < 0.05). CORT was increased from preexercise, whereas lactate increased in all conditions (*p* < 0.05). Peak force was significantly reduced from pre (439 ± 95 and 429 ± 121 *N*) to postexercise (345 ± 91 and 361 ± 94 *N*) for BASE and WARM, respectively, (*p* < 0.05). Twitch contractile duration declined (∼14%; *p* < 0.05) in all conditions along with time to peak force (∼17%, *p* < 0.05) in BASE and WARM. We conclude that OC use does not affect self‐paced cycling performance across ambient conditions with no detrimental alteration in neuromuscular performance across the menstrual cycle.

## Highlights

- Monophasic oral contraceptive use did not alter self‐paced cycling performance in either cool (20°C) or warm (32°C) conditions.
- Core temperature rose more in the heat, yet performance, exertion, and physiological strain remained stable.
- Neuromuscular strength and contractile properties were maintained across contraceptive phases and environments.
- Findings suggest oral contraceptives help preserve thermoregulatory and neuromuscular consistency, supporting reliable performance throughout the pill cycle.

## 1 Introduction

Fluctuations in the female sex hormones, estrogen, and progesterone during the normal menstrual cycle are thought to influence exercise performance (Moran et al. 1999), tolerance to heat stress (Kaciuba‐Uscilko and Grucza 2001) and muscle strength (Bennell et al. 1999). During the luteal phase, basal body temperature is elevated and thought to be due to increased levels of progesterone concentration (Hessemer and Bruck 1985; Carpenter and Nunneley 1988). Although the direct mechanism for the elevated basal body temperature is still unknown, it is thought that progesterone acts directly on temperature sensitive neurones in the preoptic area (Nakayama et al. 1975), altering the thermoregulatory set‐point based on the hormonal concentration. An elevated set point during the luteal phase may reduce exercise capacity especially when the ambient temperature is elevated (Galloway and Maughan 1997).

When exercise time to exhaustion was evaluated during the menstrual cycle in the heat (40°C, 30% relative humidity) (Tenaglia et al. 1999), exercise time was longer in the follicular compared to the luteal phase, although terminal rectal temperatures were not different but when compared based on oral contraceptive (OC) use, the menstrual phase had no effect on time to exhaustion. Notably, exercise was light, intermittent walking at 4 km/h, and it is difficult to conclude whether long duration or high intensity exercise is compromised due to altered thermoregulation during the different phases of the menstrual cycle with the use of OC.

Changes in skeletal muscle strength can vary across the ovarian cycle due to changing levels of estrogen and progesterone, affecting neuromuscular and musculoskeletal function. For example, estrogen enhances muscle contractility, reduces protein catabolism, and improves neuromuscular efficiency and tendon stiffness, which can result in improved strength during the late follicular and ovulatory phases (Lowe et al. 2010; Sarwar et al. 1996). Conversely, during the luteal phase when progesterone is highest, central nervous system excitability and motor unit recruitment can be attenuated, fatiguability is heightened, leading to reduced skeletal muscle strength (Tenan et al. 2016; Phillips et al. 1996). In addition, connective tissue laxity and fluid balance may modulate joint stability and force transmission (Herzberg et al. 2017).

The natural hormonal fluctuations of the ovarian cycle are altered by OC use by the exogenous estrogen and progestin in relatively stable concentrations resulting in suppressed ovulation and endogenous hormone variation. This hormonal stability may blunt the normal cyclical effects on neuromuscular function and muscle strength observed during the normal ovarian cycle. As such, muscle contractility, central nervous system excitability, and connective tissue stiffness variability might be stabilized (Reis et al. 1995; Elliott‐Sale et al. 2021). However, the effects of OCs may be very dependent on the type, dose, and androgenicity of the progestin component, with some formulations possibly leading to small reductions in muscle strength or hypertrophic response compared to naturally cycling women (Sung et al. 2014).

An additional consideration is the relationship between exercise, stress hormone response, and OC use. Exercise is known to increase cortisol levels in proportion to exercise intensity above a critical threshold of 50%–60% of maximal oxygen consumption (*V*O_2max_) (Viru 1992). However, during submaximal exercise, the cortisol response can be variable and if below a critical threshold cortisol values may not rise above resting values (Hill et al. 2008), although chronic training might increase the threshold for cortisol to rise. The use of OC has been shown to blunt cortisol reactivity relative to normally circulating females (Gervasio et al. 2022). Since the physiological role of cortisol includes stimulation of gluconeogenesis, mobilization of amino acids and stimulation of lipolysis (Guyton and Hall 2006), which can have a direct effect on exercise performance (Murphy et al. 2020), a blunting of cortisol reactivity with OC use might also have an effect on the physiological response that would be otherwise expected when not using OC. As far as we are aware, there are no data which have examined the cortisol response during exercise heat stress over the menstrual cycle in women taking regular OC and whether this might affect exercise and neuromuscular performance.

To this end, our hypothesis was that healthy women, using monophasic oral contraceptives, would exhibit stable exercise performance, muscle strength, and cortisol responses across the menstrual cycle due to OC hormonal stabilization. Therefore, the purpose of this study was to examine the effect of the different phases of the menstrual cycle and OC on neuromuscular properties and self‐regulated exercise in the heat.

## 2 Materials and Methods

### 2.1 Participants

Eight healthy physically active females (mean ± SD; age 20.9 ± 2.9 years; mass 67.9 ± 12.4 kg; height 1.63 ± 0.06 m; and peak oxygen consumption (*V*O_2peak_) 2.3 ± 0.42 L/min) gave written informed consent to participate in the study approved by the Ethics in Human Research Committee of the university. Participants were nonsmokers, physically active for at least an hour, three times per week, unaccustomed to exercising in the heat, not carrying any type of knee or shoulder injury, and taking a monophasic oral contraceptive pill (OC; Levlen 28; 30 μg ethinyloestradiol and 150 mg levonorgestrel and Brenda‐35 ED; and 35 μg ethinyloestradiol and 2 mg cyproterone acetate) for at least 6 months prior to exercise testing. The environmental condition was assigned according to OC phase, so that each participant was tested twice in the “active pill” phase and once in the placebo or withdrawal phase.

### 2.2 Experimental Design and Procedures

Our reasoning for the experimental design was based on the understanding that the 28 days combined monophasic OC cycle consists of 7 days of sugar pills and 21 days of constant hormone (synthetic estrogen and progesterone) concentration (Miller and Notter 2001; Sherif 1999). In a female with a natural cycle, there are fluctuations in both follicle stimulating hormone (FSH) and luteinizing hormone (LH) released from the anterior pituitary gland as well as in estrogen and progesterone released from the ovaries (Driver et al. 1996; Baker and Driver 2007). The levels of synthetic hormone in the active pills prevent events of the natural ovarian and uterine cycles, including follicle maturation, ovulation, LH surge, increase in core body temperature, and FSH dip from occurring (Baker et al. 2020). Thus, it is believed that this eliminates the hormone fluctuations across the 28 days although recent evidence suggests that this is not as definitive as originally thought (Rodriguez et al. 2024). The stimulus for uterine shedding in the natural cycle is the drop in estrogen and progesterone if fertilization does not occur and as the corpus luteum degenerates. As such, OC use has the effect of stabilizing the *T*_c_ over the course of the menstrual cycle. However, it should be noted that hormonal stabilization is expected to result in a higher *T*_c_ of ∼0.5°C compared to women not taking a dose of OC. Importantly, monophasic OC can mask the natural menstrual cycle due to the action of the “active” hormones (Sunderland and Nevill 2003).

The first visit to the laboratory was for a familiarization session to obtain a baseline measure of *V*O_2peak_, and for the familiarization with the cycling equipment, electromyography (EMG) and muscle activation procedures to be used during testing.

A critical consideration for our experimental design was ecological validity. Unlike many studies that artificially standardize menstrual cycle phase by repeatedly testing in the same phase across multiple months, our approach recognizes that athletes cannot schedule competitions around optimal hormonal conditions. Therefore, examining the effects of oral contraceptive use within the context of a real uninterrupted menstrual cycle yields evidence that is both scientifically robust and practically relevant. This design reflects the actual constraints faced by female athletes and provides findings that can be translated directly to real‐world performance scenarios. Therefore, the order and rationale for the experimental design within the menstrual cycle was as follows: Testing was performed on days 1, 8, and 18 within a single OC treatment course. Day 1 of the treatment course refers to the first day of withdrawal bleeding, marking the start of the treatment course and the ingestion of a placebo (sugar) pill, where serum estradiol and progesterone are at their lowest. Testing on day 1 was performed at 20°C and served as the baseline condition (BASE). Days 8 and 18 of the treatment course involved the ingestion of active synthetic hormone pills. These days were selected as they correspond to the timing of the mid‐luteal and mid‐follicular phases of the natural cycle, respectively, where fluctuations in serum estradiol and progesterone, respectively, normally occur.

However, during the OC treatment course, hormone fluctuations associated with a natural cycle are substantially blunted and serum estradiol and progesterone between days 8 and 18 days are highly comparable. As such, testing on day 8 was performed at 20°C (COOL); whereas testing on day 18 was performed at 32°C (WARM). Day 18 was specifically chosen for the 32°C condition to provide a robust context for examining the effects of oral contraceptive use on exercise performance in the heat. This time point aligns with the mid‐luteal phase of the natural menstrual cycle when hormonal fluctuations are typically associated with elevated body temperature and altered exercise performance. By selecting day 18 for the warm condition, this study maximizes its ability to explore how OC within a single cycle influence these physiological responses under thermally challenging conditions. Furthermore, since training and competition occur in different temperatures during the menstrual cycle, adding a heat stress condition on day 18 introduces and ecologically valid component.

Participants were asked to report to the laboratory rested and fasted for a minimum of 3 h prior to testing. Additionally, they were asked to refrain from exercising and consuming alcohol or caffeine in the 12 h preceding testing. A 24 h food diary was maintained for the day prior to the first test so that individuals could follow similar eating patterns on the days immediately prior to subsequent tests. Nude mass was measured after voiding. After which, an indwelling venous cannula (BD Saf‐T‐Intima, Utah) was introduced into a superficial forearm vein to allow for repeated blood sampling throughout the testing session. Site preparation for electrode or thermistor attachment consisted of identifying the correct site, shaving the hair, abrading the outer layer of the epidermal cells, and cleaning with an alcohol swab. After attachment, all electrodes and thermistors were firmly taped to the skin to minimize sweat interference.

Following a 2 min warm up on a stationary bike at self‐selected intensity, the level of maximal voluntary activation (VA), maximal voluntary force (MVC), surface EMG, and potentiated twitch contractile properties for the right knee extensors and right elbow flexors were performed on a KinCom isokinetic dynamometer (Model 125H; Chatanooga Group Inc., Hixon, Tennessee, USA).

Participants then commenced a cycling protocol under their allocated testing condition. Immediately following the cycle testing protocol participants repeated the neuromuscular testing. Testing sessions were separated by 7–10 days, depending on when in the OC cycle testing began. All cycle testing was performed inside a climate chamber in either a moderate (20.0 ± 0.5°C) or warm (32.0 ± 1.1°C) ambient environments with relative humidity set at 48 ± 3% and 66 ± 2%, respectively. Regardless of ambient temperature, participants were permitted to consume plain water *ad libitum* throughout the testing sessions.

### 2.3 Peak Oxygen Uptake Test

The *V*O_2peak_ test was conducted on an Avanti road bicycle (Corsa Pro Elite Series) mounted on a Tacx cycle ergometer (Cosmos, Model T1970, Tacx bv, Netherlands) with the front wheel stabilized by a Tacx Skyliner (Model T1979, Tacx bv, Netherlands). After a free paced warm up for 2 min, the test began at a workload of 50 W (W) and then increased by 50 W every 2 min until volitional termination. Throughout the test participants remained seated but were permitted to change gears and/or cadence as desired. The gas analyzers (True One 2400 Metabolic Measuring System, Parvo Medics, USA) were calibrated with a 3 L calibration syringe (Hans Rudolph Inc., USA) and reference samples of oxygen (O_2_) (16.01%) and carbon dioxide (CO_2_) (3.98%) (Airgas, Puritan Medical Products, Overland Park, KS, USA). Participants breathed through a one‐way valve (Hans Rudolph, USA) with the air transported via a 3.5 m hose through a pulmonary function filter (Creative Biomedics Inc., USA) into a mixing chamber where gases were analyzed for O_2_ and CO_2_.

### 2.4 Cycling Trials

Cycle testing was performed with the same apparatus used during the *V*O_2peak_ test. Data for each experimental session were recorded through Fortius Software for Cosmos Ergometer (v1.29, Tacx bv, Netherlands).

Following the preexercise neuromuscular testing, participants commenced a 30 min fixed‐intensity cycle period. This was performed at 50% *W*_max_ calculated from the previous *V*O_2peak_ test programmed at the beginning of each trial through the Fortius Software to ensure the fixed intensity was maintained throughout. Gear settings were kept constant throughout the entire 30 min. Subsequently, a 3 min rest was provided in the climate chamber to allow for collection of blood samples. Immediately following the 3 min rest period, the 30 min self‐paced time trial (TT) commenced. During both the fixed intensity and TT, 30 s sprints were completed at the 9, 19, and 29 min mark. This protocol was chosen to reflect ecologically appropriate exercise given that the inclusion of sprints and self‐paced periods have been shown to be more reliable and produce smaller variations in performance between trials compared with fixed‐intensity protocols (McLellan et al. 1995; Marino et al. 2002).

For each TT, the participant was instructed to cycle as far as possible in the 30 min. Participants were permitted to change the bicycle gears as desired throughout the TT. No feedback was provided to the participant during any part of the trial apart from a countdown to the next sprint given at 2 min, 30, and 10 s prior to the commencement of the sprint. The participants were given strong verbal encouragement by the researchers during the sprints. During the sprints, EMG was recorded from three sites on the right thigh (EMGworks Signal Acquisition and Analysis Software version 3, Delsys, USA), described in detail subsequently. Upon completion of the 30 min TT, a final blood sample was taken before the participant exited the climate chamber after which neuromuscular data were reassessed in the same order as performed prior to cycle testing. The participant was disconnected from the apparatus and a final nude body mass was recorded to estimate total body sweating. Distance covered in km, average, and peak cadence in rpm, average and peak speed in km/h, and average and peak power (W) recorded from the Tacx software at 5 min intervals throughout the cycling protocol.

### 2.5 Blood Sampling and Analysis

Upon reporting to the laboratory, the subject rested in a chair while a 20‐gauge cannula was inserted into a superficial forearm vein in the left arm and covered with an adhesive dressing. Blood samples were drawn immediately following the cannula set up, immediately following the fixed‐intensity cycling protocol and upon completion of the 30 min time trial. The line was kept patent by flushing with 0.9% sodium chloride after each blood draw and approximately every 5 min. After drawing off the sodium chloride into a 3 mL syringe, blood samples were divided into precooled K_3_EDTA tubes for determination of cortisol (COR). The tubes were inverted six times and centrifuged immediately at 4500 rpm in a refrigerated centrifuge for 10 min. Separated plasma was placed into 1 mL aliquots and frozen at −80°C until further analysis for COR. In addition, a 0.5 mL aliquot of whole blood was drawn into a syringe for determination of lactate (La^−^) (ABL800 Flex Radiometer, Copenhagen).

Before analysis the serum was thawed to room temperature and mixed gently via inversion. Plasma estradiol, progesterone, and cortisol concentrations were assessed by a solid‐phase two‐site chemiluminescent immunometric assay with detection limits of 25 pg.mL^−1^, 0.2 ng.mL^−1^, and 5.5 nmol.L^−1^, respectively (Immulite 2000, Diagnostic Products Corporation, Los Angeles, USA). To avoid interassay variations, all samples for each participant were assayed in the same assay run. Serum hormone concentrations were not corrected for plasma volume shifts (Bird et al. 2006), thus all analyses were performed on hormone values based on actual measured circulating concentrations. Unfortunately, both plasma estradiol and progesterone values were either not within the detectable range or some values well above the normal range. As such, the high variability did not permit any further meaningful analysis. This result is not unusual and has been previously reported (Fehring et al. 2006; Roos et al. 2015; Direito et al. 2013) and could be due to any number of factors such as menstrual cycle phase, contraceptive use, individual metabolism, and handling of the samples. Intraassay reliability was calculated as SD/mean*100 which for COR was calculated to be 32.42%.

### 2.6 Thermoregulatory Measures

Four hours prior to reporting to the laboratory, participants were instructed to ingest a telemetry pill for determination of intestinal temperature as an index of *T*_c_ which was recorded at 5 min intervals throughout each testing session (Vital Sense, Mini Mitter Company Inc., USA). Upon arrival at the laboratory, participants had four skin thermistors fastened to four sites as previously described and a mean skin temperature (*T*_s_) (Ramanathan 1964) calculated at 5 min intervals throughout the cycling protocol.

### 2.7 Perceptual Measures

The rating of perceived exertion (RPE) 1–10 (Borg 1990) and perception of thermal sensation (Gagge et al. 1967) were recorded at 5 min intervals from the beginning of the cycling protocol until the completion of the final sprint.

### 2.8 Heart Rate

Heart rate was continuously monitored and recorded at 5 min intervals (FS1; Polar Electro Oy, Kempele, Finland) during the cycling protocol. In order that no feedback was available to participants, the HR receiver was attached to the heart rate monitor strap on the participant's back.

### 2.9 Neuromuscular Measurements

#### 2.9.1 Participant Setup

For all assessments, participants were seated on an isokinetic dynamometer (Kin‐Com, Model 125H; Chattanooga Group, Chattanooga, TN). The upper body was supported by the chair back, and the hips were positioned at a 100° angle (0° = full extension), secured with a waist strap. Arms remained crossed over the chest to minimize upper body contribution during testing.

*Knee Extensor Setup:* Participants' knees were flexed at 90° (0° = full extension). The dynamometer's axis was aligned with the lateral femoral epicondyle, and the lower leg was strapped to the lever arm ∼1 cm proximal to the lateral malleolus.

*Elbow Flexor Setup:* The right arm was supported with the elbow flexed at 65° (0° = full extension). The dynamometer axis aligned with the lateral epicondyle of the humerus, with the forearm secured to the lever arm at the radial styloid process. The order of limb testing and contraction type (voluntary or evoked) was counterbalanced across trials.

### 2.10 Muscle Activation and Data Acquisition

Superimposed maximal voluntary contractions and twitch assessments were performed on the right limbs under isometric (ISO) conditions using a constant‐current stimulator (DS7AH; Digitimer; Welwyn Garden City, Hertfordshire, UK). Single square‐wave pulses (200 μs width, 250–450 mA current) were delivered to elicit muscle responses. Activation of the knee extensors was achieved using two reusable 2” × 5” rectangular gel electrodes (PALS Platinum, CA, USA) placed 1 cm apart over the femoral nerve on the anterior aspect of the proximal thigh, just below the inguinal fold. Activation of the elbow flexors was achieved using two disposable 20 mm diameter Ag/AgCl electrodes (Medi‐Trace Mini 100 Snap, Kendall, Chicopee, MA, USA) placed over the proximal and distal ends of the biceps brachii muscle belly. This configuration was used to stimulate the intramuscular nerve branches of the musculocutaneous nerve. For both limbs, stimulus intensity during testing was progressively increased until a plateau was observed in both the resting twitch amplitude and the maximum compound muscle action potentials (*M*_max_) recorded from the biceps brachii or the vastus medialis and vastus lateralis. The intensity was then increased by a further 25% to ensure supramaximal stimulation. Activation of the antagonist muscles was also monitored to ensure minimal co‐contraction during testing. Before electrode placement, the skin was prepared by shaving any hair and cleansing the area with isopropyl alcohol. The borders of each electrode were marked on the skin to maintain consistent placement across all trials. All data were collected using a custom signal acquisition system where analog force, surface EMG, and muscle stimulator trigger signals were routed through a terminal block that performed A/D conversion at 16‐bit resolution (BNC‐2100 National Instruments, Austin, TX) connected to a host acquisition device (PXI1024, National Instruments, Austin, TX) that synchronously sampled data at 2000 Hz using custom software (LabVIEW 8.0, National Instruments, Austin, TX).

### 2.11 Superimposed Maximal Voluntary Contractions and Voluntary Activation Levels

During all testing procedures, participants were instructed to initiate a maximal voluntary contraction as rapidly as possible and to sustain maximal effort until prompted to relax, typically within 3–4 s. A superimposed stimulus was manually delivered 1–2 s after MVC initiation, coinciding with the plateau of peak force. Within 5 s following the superimposed contraction, a second stimulus was administered while the muscle was at complete rest to assess twitch contractile properties. Each assessment included four trials. For preexercise testing, a 30‐s rest interval was provided between trials, whereas postexercise trials were conducted consecutively with minimal delay. Voluntary activation levels during the superimposed maximal voluntary contraction were calculated using the twitch interpolation technique (Herbert and Gandevia 1999). Peak superimposed force (SIMVC) was defined as the highest force value recorded within 50–150 ms following stimulus delivery. Voluntary peak force (MVC) was quantified as the mean force generated during the 50 ms immediately preceding the stimulus. Interpolated twitch force (ITF) was subsequently determined as the SIMVC minus MVC and was calculated to four decimal places. Voluntary activation levels were then determined by expressing the ITF as a percentage of the peak potentiated twitch force (Pf) obtained at rest; VA (%) = [1−(IFT/Pf)] × 100. All trials were assessed with the trial yielding the highest VA used for subsequent analysis. These procedures were performed using MATLAB Software (R2009b 7.9.0.529, Mathworks Inc., Natick, USA).

### 2.12 Potentiated Evoked Twitch Contractile Properties

Force‐time curves from the potentiated evoked twitch contractions were averaged across all trials with mean data used to determine the following: (1) peak potentiated twitch force (Pf; the highest force value obtained during the resting evoked contraction); (2) rate of force development (RFD; the mean tangential slope of the twitch force‐time curve between the onset of force development and Pf); (3) time to peak force (TPf; time from evoked force onset to Pf); and (4) rate of relaxation (RR; the mean tangential slope of the twitch force‐time between Pf and ½RT); half‐relaxation time (½RT; the time required for Pf to decline by half) and contraction duration (CD; TPf plus ½RT). These procedures were performed using MATLAB Software (R2009b 7.9.0.529, The Mathworks Inc., Natick, USA).

### 2.13 Surface Electromyography

Surface EMG signals were captured using single differential sensors (model DE.2.1; bar configuration: 1 × 10 × 10 mm; bandwidth: 20–450 Hz; Delsys Inc., Boston, MA) and amplified (Bagnoli‐4, Delsys Inc., Boston, MA) with a common‐mode rejection ratio of 92 dB and gains of 100 V/V and 1000 V/V for evoked and voluntary efforts, respectively. For the lower limb, EMG sensors were placed on the vastus lateralis, vastus medialis, and biceps femoris. For the upper limb, sensors were positioned on the biceps brachii and lateral head of the triceps brachii. All sensor placements followed SENIAM recommendations (Hermens et al. 2000). The borders of each electrode were marked on the skin to maintain consistent placement across all trials. All sensors were securely fixed to the skin using micropore tape. For processing, EMG signals were first zero‐averaged to remove any DC bias and bandpass filtered between 20 and 450 Hz using a second‐order Butterworth filter with Mmax determined as the peak‐to‐peak amplitude of the filtered signal.

### 2.14 Statistical Analyses

A priori power calculations were conducted using G*Power (G*Power 3.1.2, Franz Faul, Germany), which indicated 8 participants were needed with power set 0.80. Repeated measures ANOVAs were used to determine differences between environmental and OC conditions in cycling performance, neuromuscular properties, or biochemical markers. When interactions or main effects achieved statistical significance, Tukey's HSD *post hoc* test was used to identify differences between means. Statistical significance was set at *p* < 0.05. Data are reported as mean ± SD. Where applicable, results are reported with *F*‐values, degrees of freedom (df), exact *p* values, and effect sizes as partial eta‐squared (*η* ^2^). The *η* ^2^ values are reported as a measure of effect size for repeated measures ANOVA where *η* ^2^ represents the proportion of variance in the dependent variable explained by a given effect, accounting for other variables in the model. Effect sizes were interpreted according to conventional benchmarks as previously suggested (Cohen 1988) as either small (*η* ^2^ ∼ 0.01), medium (*η* ^2^ ∼ 0.06), and large (*η* ^2^ ≥ 0.14). Data analyses that resulted in no significant main effects or interactions are also reported with exact *p*‐values and corresponding effect sizes.

## 3 Results

### 3.1 Cycling Performance

Table 1 provides the various parameters measured during both fixed‐intensity and self‐paced exercise periods for each condition.

**TABLE 1:** Fixed intensity and self‐paced cycling performance measures.

	Fixed intensity			Time trial		
	BASE (20°C)	COOL (20°C)	WARM (20°C)	BASE (20°C)	COOL (20°C)	WARM (20°C)
Total distance (km)	15.3 ± 2.2	15.7 ± 3.7	15.9 ± 3.1	11.1 ± 1.1	10.8 ± 1.2	10.9 ± 1.1
Mean power output (W)	116.0 ± 19.6	116.0 ± 19.6	116.0 ± 19.6	127.4 ± 12.3	125.8 ± 15.5	128.4 ± 15.0
Peak power output sprints (W)	116.0 ± 19.6	116.0 ± 19.6	116.0 ± 19.6	362.5 ± 85.7^a^	349.9 ± 97.4^a^	364.9 ± 65.1^a^
Mean speed (km.h^−1^)	30.1 ± 4.5	30.9 ± 7.2	31.1 ± 6.0	22.0 ± 2.0	21.2 ± 2.4	21.4 ± 1.9
Max speed sprints (km.h^−1^)	57.6 ± 4.0^b^	57.7 ± 3.4^b^	57.8 ± 6.0^b^	37.0 ± 4.6^b^ ^,^ ^c^	34.2 ± 3.5^b^	40.9 ± 7.0^b^ ^,^ ^c^
Mean cadence (rpm)	75.0 ± 11.2	71.0 ± 11.1	71.0 ± 10.9	69.0 ± 8.2	70.0 ± 8.9	67.0 ± 12.6
Max cadence sprints (rpm)	132.0 ± 11.9^b^	124.0 ± 6.0^b^	130.0 ± 12.6^b^	115.0 ± 6.6^b^	110.0 ± 11.5^b^	116.0 ± 7.8^b^

^a^ *p* < 0.05 significant increase in speed during sprints compared to mean in self‐paced cycling within all conditions.

^b^ *p* < 0.05 significant increases in speed and cadence from mean to maximum during intermittent sprints within both trials for all conditions.

^c^ *p* < 0.05 significant increase in maximum speed in self‐paced cycling in CON and WARM, compared to COOL with the self‐paced trials.

### 3.2 Fixed Intensity

Power output during fixed‐intensity cycling was programmed manually prior to exercise commencing for each participant; therefore, there were no differences in distance cycled, mean cadence, or speed between conditions. PO was equivalent to 50% *V*O_2max_ and for the group was 116 ± 19.6 W. However, maximum cadence (*F* (3, 21) = 43.2, *p* = 0.001, and *η* ^2^ = 0.86) and speed (*F* (3, 27) = 25.9, *p* = 0.001, and *η* ^2^ = 0.92) during the sprints were significantly higher than mean values within all conditions.

### 3.3 Self‐Paced

There were no significant differences in distance cycled, mean cadence, power output, and speed or maximum cadence, power output, and speed between environmental or contraceptive phase. However, maximal cadence, power output, and speed during the sprints were all significantly higher than mean values within all conditions. Speed during the maximal sprints was faster in BASE and WARM conditions compared with the COOL (*F* (2, 18) = 4.81, *p* = 0.02, and *η* ^2^ = 0.35) by ∼ 2.8 km.h^−1^ and ∼ 6.7 km.h^−1^, respectively (*p* = 0.001). Maximum power output was higher over time (*F* (2, 18) = 103.9, *p* = 0.0001, and *η* ^2^ = 0.96) across each condition but was not different (*F* (2, 18) = 0.29, *p* = 0.75, and *η* ^2^ = 0.03) between conditions.

### 3.4 Thermoregulatory Responses

#### 3.4.1 Core Temperature

**FIGURE 1:**
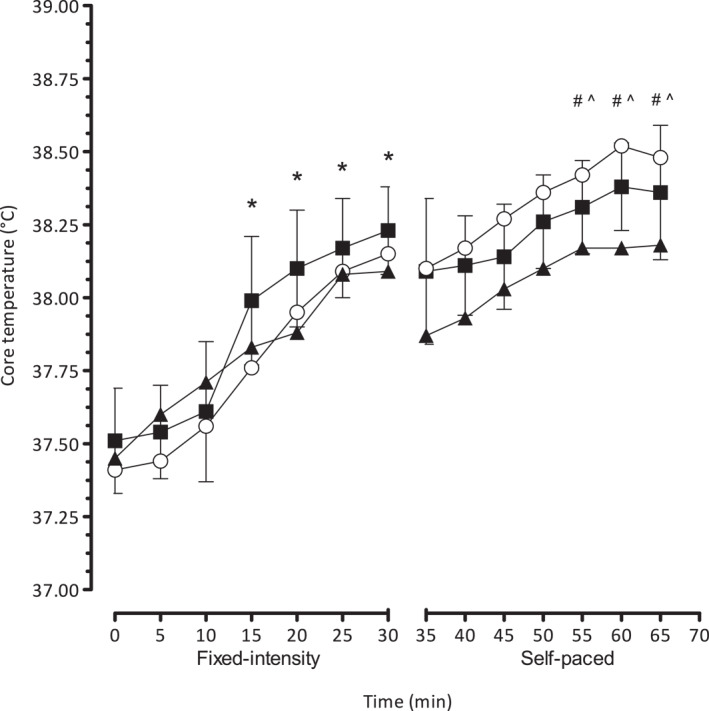
Core temperature response during fixed‐intensity (0–30 min) and self‐paced time trial performance (35–65 min). Conditions: BASE (▲) and COOL (■) are 20°C and WARM (〇) is 32°C ambient temperatures, respectively. *p < 0.05 significant increases from baseline values in all conditions, ^#^ *p* < 0.05 significant differences between CON and WARM. ^*p* < 0.05 significant increase from the commencement of self‐paced cycling in all conditions.

The *T*_c_ response for both fixed intensity and self‐paced exercise periods for each condition is shown in Figure 1.

#### 3.4.2 Fixed Intensity

During fixed intensity cycling exercise, *T*_c_ increased significantly from baseline (*F* (2, 6) = 25.5, *p* = 0.004, and *η* ^2^ = 0.92). By the end of the 30 min, *T*_c_ had reached ∼38.1°C in BASE, ∼38.3°C in COOL, and ∼38.2°C in WARM. Cycling in COOL was significantly higher than BASE (*p* = 0.04).

#### 3.4.3 Self‐Paced

Following the commencement of self‐paced cycling, *T*_c_ continued to rise significantly in WARM, reaching approximately 38.5°C and also in BASE and COOL, reaching values of ∼38.2°C and ∼38.4°C, respectively (*F* (2, 6) = 78.3, *p* = 0.0001, and *η* ^2^ = 0.91). In the final 15 min of self‐paced cycling, *T*_c_ was significantly higher in WARM than in BASE (*p* = 0.004).

### 3.5 Skin Temperature

The *T*_s_ increased significantly at all time points in WARM and was significantly higher compared with BASE and COOL (*F* (2, 6) = 83.4, *p* = 0.002, and *η* ^2^ = 0.98). There was no significant difference in *T*_*s*_ between BASE and COOL (*p* = 0.45).

#### 3.5.1 Heart Rate

**FIGURE 2:**
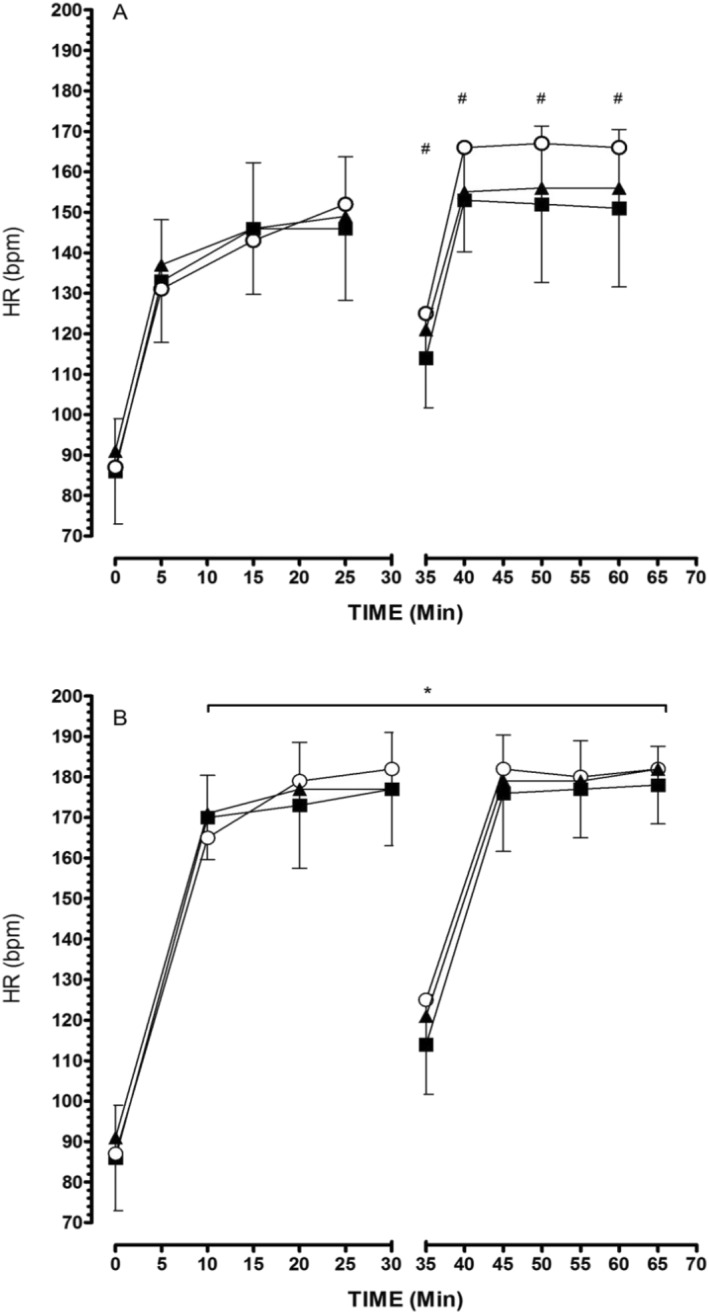
Heart rate responses during low intensity efforts (A) and sprints (B) over the entire trial. BASE (▲) and COOL (■) are 20°C and WARM (〇) is 32°C ambient temperatures, respectively. *p < 0.05 significant difference between low intensity and sprints. HR during exercise was significantly higher than 0 min ^#^ *p* < 0.05 significant difference between low intensity HR for COOL vs WARM.

Figure 2 shows the mean HR responses from each of the fixed intensity and self‐paced exercise periods for each condition. HR was significantly increased from preexercise (∼88 beats/min) in all conditions and exercise periods. The HR was predictably higher during sprints in both exercise periods for all conditions. During the fixed intensity sprints, HR typically reached ∼ 174 beats/min across all trials. These values were slightly higher during the self‐paced exercise period (∼178 beats/min for both BASE and COOL) and ∼181 beats/min for WARM) but were not statistically different (*F* (1, 8) = 2.34, *p* = 0.165, and *η* ^2^ = 0.22 (*p* > 0.05). Following each of the sprints, the HR was reduced to ∼ 147 beats/min across the fixed intensity period. HR following the sprints during self‐paced efforts were significantly lower in BASE and COOL (∼153 beats/min) compared with WARM (∼166 beats/min; *F* (1, 8) = 170.9, *p* = 0.0001, and *η* ^2^ = 0.95).

#### 3.5.2 Perceived Exertion

Table 2 includes the mean RPE responses from each of the fixed‐intensity and self‐paced exercise periods for each condition. As no statistically significant differences were detected among conditions, only the means for sprints and low intensity efforts have been included for clarity. Similar to the HR response, the RPE during the sprints increased from the start of exercise to reach values of 5.2–5.6 then decreasing to ∼ 3.5 (*F* (2, 6) = 90.3, *p* = 0.0001, and *η* ^2^ = 0.22) during the low intensity efforts in the fixed intensity exercise periods in all trials. In self‐paced cycling periods, the RPE during the sprints increased from the start of the section to ∼7 then decreasing to ∼5–5.7 during the low intensity efforts in BASE and COOL (*F* (2, 6) = 79.1, *p* = 0.0001, and *η* ^2^ = 0.99) and reached ∼8 then decreased to ∼6.7 in the WARM condition (*p* = 0.001).

**TABLE 2:** Rating of perceived exertion (RPE) during the low intensity effort and sprints in fixed intensity and self‐paced exercise periods for each trial.

	Intensity	BASE (20°C)	COOL (20°C)	WARM (32°C)
Fixed‐intensity	Low RPE	3.5 ± 1.6	3.7 ± 1.3	3.6 ± 1.6
	Sprints RPE	5.2 ± 1.6^a^	5.3 ± 1.7^a^	5.6 ± 2.1^a^
Self‐paced	Low RPE	5.3 ± 1.8	5.7 ± 1.6	6.7 ± 1.8^b^
	Sprints RPE	7.1 ± 1.4^a^	7.3 ± 1.2^a^	8.1 ± 1.5^a^, ^b^

^a^ *p* < 0.05 significant increase in RPE from low to high intensity efforts within all trials and conditions.

^b^ *p* < 0.05 higher RPE in both low and high intensity efforts compared with CON.

#### 3.5.3 Thermal Sensation

Thermal sensation was significantly increased from preexercise compared across all time points for both fixed‐intensity (*F* (2, 6) = 11.1, *p* = 0.018, and *η* ^2^ = 0.94) and self‐paced (*F* (2, 6) = 65.72, *p* = 0.001, and *η* ^2^ = 0.99) cycling in all conditions. Further, thermal sensation was higher in the WARM condition compared to BASE and COOL conditions (*p* = 0.004) at all time points for both fixed‐intensity and self‐paced cycling; however, there were no differences between the BASE and COOL conditions (*p* = 0.10).

### 3.6 Neuromuscular Responses

#### 3.6.1 Maximal Voluntary Activation and Isometric Contractions

In all conditions, postexercise MVC was maintained for forearm flexors (*F* (2, 12) = 0.37, *p* = 0.70, and *η* ^2^ = 0.06) and decreased for the leg extensors (*F* (2, 12) = 5.86, *p* = 0.0.47, and *η* ^2^ = 0.45) compared to the preexercise MVC (Table 3). The decrease postexercise was significant for BASE and WARM (*p* = 0.047). There were no significant differences in VA% of the forearm flexors (*F* (1, 6) = 0.31, *p* = 0.0.60, and *η* ^2^ = 0.05) or the knee extensors (*F* (1, 6) = 0.43, *p* = 0.53, and *η* ^2^ = 0.06) during pre and postexercise MVC among conditions.

**TABLE 3:** Maximal voluntary contraction (MVC) and voluntary activation (VA) of the forearm flexors (FF) and leg extensors (LE) pre and postcycling exercise.

		MVC (*N*)		VA%	
		Pre	Post	Pre	Post
BASE (20°C)	FF	160.1 ± 33.0	145.9 ± 30.5	100.9 ± 1.7	102.3 ± 2.8
	LE	438.9 ± 95.0	345.4 ± 91.2^a^	85.9 ± 11.8	87.9 ± 8.2
COOL (20°C)	FF	166.1 ± 32.7	162.6 ± 23.8	101.0 ± 3.1	103.0 ± 4.4
	LE	430.2 ± 130.5	387.0 ± 112.4	82.9 ± 15.8	81.6 ± 9.9
WARM (32°C)	FF	161.5 ± 34.6	155.8 ± 42.4	100.1 ± 2.8	100.8 ± 1.9
	LE	429.4 ± 121.1	360.6 ± 94.4^a^	85.6 ± 16.7	81.1 ± 11.7

^a^ *p* < 0.05 MVC for the leg extensors postexercise were significantly lower than preexercise MVC within the CON and WARM trials.

#### 3.6.2 Potentiated Evoked Twitch Contractile Properties

There were no significant differences in the contractile properties of the biceps postexercise in any condition (*F* (1, 6) = 2.08, *p* = 0.16, and *η* ^2^ = 0.26). Table 4 includes mean data for potentiated twitch contractile properties for the leg extensors. The contractile properties for leg extensors resulted in significant decreases postexercise for TPf in BASE and WARM only and contraction duration in all conditions compared with preexercise (See Table 4 for statistical outcomes).

**TABLE 4:** Leg extensor potentiated evoked twitch contractile properties preexercise and postexercise each condition.

		Pf (*N*)	RFD mean (N.s^−1^)	TPf (ms)	½ RT (ms)	CD (ms)
BASE (20°C)	Pre	190 ± 51	907.8 ± 277.9	103 ± 14	87 ± 29	190 ± 30
	Post	145 ± 31	852.4 ± 151.3	83 ± 11^a^	75 ± 5	159 ± 10*
COOL (20°C)	Pre	163 ± 21	806.0 ± 173.6	101 ± 14	96 ± 31	196 ± 28
	Post	141 ± 30	790.3 ± 214.5	89 ± 14	79 ± 10	168 ± 14^b^
WARM (32°C)	Pre	149 ± 48	765.4 ± 256.9	96 ± 7	91 ± 10	187 ± 8
	Post	147 ± 45	861.6 ± 219.0	83 ± 10^a^	80 ± 15	163 ± 17^b^

Abbreviations: CD, contraction duration (*F* (1, 5) = 19.01, *p* = 0.007, and *η* ^2^ = 0.80); Pf, peak force (*F* (1, 6) = 3.10, *p* = 0.13, and *η* ^2^ = 0.34); RFDmean, mean rate of force development (*F* (1, 5) = 0.74, *p* = 0.43, and *η* ^2^ = 0.13); TPf, time to peak force (*F* (1, 6) = 14.0, *p* = 0.01, and *η* ^2^ = 0.70); ½ RT, half relaxation time (*F* (1, 6) = 0.45, *p* = 0.53, and *η* ^2^ = 0.07).

^a^ *p* = 0.01 decrease in TPf from pre to postexercise.

^b^ *p* = 0.007 decrease in CD from pre to postexercise.

#### 3.6.3 Biochemical Markers

**FIGURE 3:**
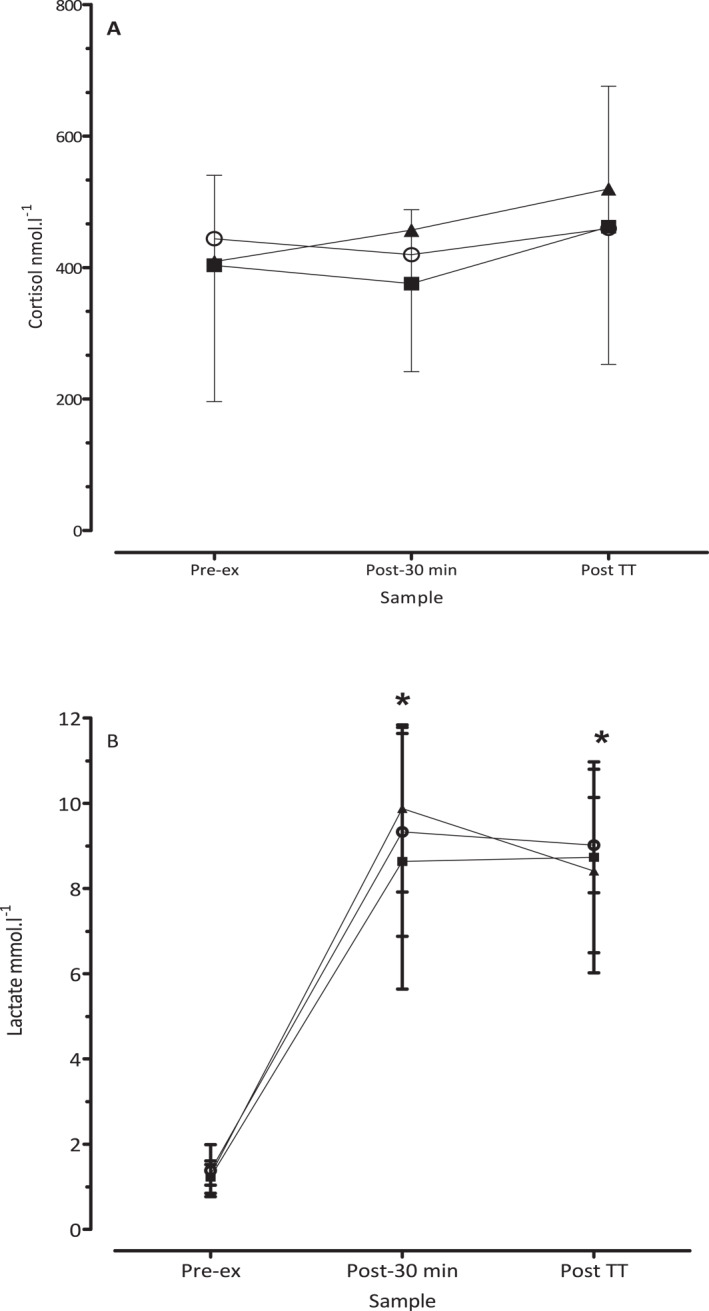
Cortisol (A) and lactate (B) responses preexercise (Pre‐ex), end of the 30 min fixed intensity cycle (Post‐30 min), and at the end of the 30 min time trial (Post TT). *p < 0.05 significantly higher lactate compared with pre‐exercise. BASE (▲) and COOL (■) are 20°C and WARM (〇).

Cortisol increased from preexercise to postexercise in all conditions and is shown in Figure 3, although these differences were not significant (*F* (2, 14) = 0.15, *p* = 0.71, and *η* ^2^ = 0.02). Blood lactate increased from preexercise compared with both post fixed‐intensity and post self‐paced exercise periods across all conditions (*F* (2, 14) = 653.6, *p* = 0.0.0001, and *η* ^2^ = 0.989).

## 4 Discussion

### 4.1 Self‐Paced Performance

The novel finding of the present study is that irrespective of environmental conditions and OC pill use, self‐paced cycling performance was not different throughout the menstrual cycle in different environmental temperatures. Our data indicate that these moderately active women achieved consistent total distance and mean power outputs across trials during both fixed intensity and self‐paced exercise periods lasting 30 min each. As far as we are aware, no studies have reported similar consistencies under the conditions of the present study. Previous studies have generally evaluated the potential effect of the menstrual cycle on exercise time to exhaustion (Hessemer and Bruck 1985; Pivarnik et al. 1992; Williams and Krahenbuhl 1997), all of which have typically reported no significant change in exercise time. Although these studies, but not all (Hall Jurkowski et al. 1981), show that exercise tolerance ranging from 70% to 90% of *V*O_2max_ is not altered during the menstrual cycle, a shift toward increased cardiovascular and thermoregulatory strain was evident during the mid‐luteal phase of the trial.

Table 1 shows that maximum speed during the self‐paced sprints was significantly reduced in COOL. Although this specific result is difficult to attribute directly to either the environmental conditions or OC use per se, we do note that a reduction in maximum speed occurred in COOL during the mid‐luteal phase of the menstrual cycle. According to previous studies, this is typically when higher RPE, cardiovascular, and thermoregulatory strain expected to occur (De Souza et al. 1990). Given our findings related to exercise performance, we can only conclude that menstrual phase and OC use is of no significant consequence for moderately active women in ambient temperatures ranging from 20°C–32°C.

### 4.2 Thermoregulatory Responses

The present experimental paradigm provides a snapshot of the thermoregulatory responses throughout the menstrual cycle (Figure 1). During the initial 30 min of fixed intensity exercise, *T*_c_ increased at the same rate across all conditions and was significantly higher at 15–30 min. This observation is particularly notable when compared to the subsequent 30 min of self‐paced cycling when there was a clear separation of *T*_c_ response relative to the experimental condition. Although the rate of rise was similar among conditions with values becoming higher during the last 15 min of the trial compared to the commencement of the self‐paced section, the BASE *T*_c_ remained lower when compared with WARM. This effectively meant that *T*_c_ values for the COOL remained relatively similar between that of the BASE and WARM (Figure 1). Since the experimental design allowed us to test the effect of OC during the mid‐luteal and mid‐follicular phases of the menstrual cycle, it is possible that the *T*_c_ response was influenced to some extent by the hormonal fluctuations even though the active pill phase supposedly diminishes direct influence on thermoregulation. Normally, during the luteal phase, body temperature is purportedly ∼0.3°C higher and either remains higher or increases more so during exercise by up to ∼0.6°C compared to the follicular phase (Pivarnik et al. 1992). However, we did not observe any differences in *T*_c_ preexercise, which suggests that the OC effectively masked the potential elevated body temperature in the luteal phase at least during the fixed intensity section of the trial. Thereafter, during the self‐paced period, the *T*_c_ during the mid‐luteal phase was significantly elevated over the remaining 15 min compared to BASE. Since total distance was 11.1 ± 1.1 km in BASE versus 10.9 ± 1.1 km in WARM, we conclude that the menstrual cycle, whilst taking OC in these women did not influence exercise even though *T*_c_ was ∼0.3 higher in WARM by the end of the trial. The observation that *T*_c_ in WARM was higher over the final 15 min of self‐paced exercise compared with BASE is curious since commencing *T*_c_ were similar for each condition. The reasons for the elevated *T*_c_ in the final stages of exercise are not clearly evident but could be due to at least two factors. First, as shown in Table 1, the maximum speeds achieved during the WARM time trial were 11% and 19.8% higher compared with BASE and COOL, respectively. This additional effort might partly explain the higher heat accumulation leading to a higher *T*_c_. Second, even though OCs suppress natural hormonal fluctuations, heat accumulation toward the end of exercise might have been augmented due to a shift to a higher threshold for sweating and reduced skin blood flow with the exogenous progestin (Charkoudian and Stachenfeld 2016; Stachenfeld et al. 2000).

Although we cannot thoroughly explain why the added heat load did not reduce self‐paced performance, Table 1 also shows that maximum speed was significantly higher in both BASE and WARM compared with COOL which could partly account for the maintained performance during higher *T*_c_, whereby pacing was adjusted in order that performance not be adversely compromised (Tatterson et al. 2000; Marino 2004; Marino et al. 2004). This was also confirmed by the HR response in the self‐paced section. A higher HR in WARM compared with the COOL (Figure 2) along with relatively higher RPE (Table 2) and overall higher lactate (Figure 3), indicate that participants gave a conscious effort in the heat, albeit with higher cardiovascular strain in the warmer condition for a similar performance outcome.

### 4.3 Cortisol and Stress Response

It is also evident that the stress response as measured by cortisol was relatively uniform from preexercise, post 30 min of fixed intensity and post time trial (Figure 3A). The expectation is that the cortisol response with OC use often results in higher total cortisol levels due to the elevated levels of cortisol‐binding globulin which binds to cortisol, making less of it “free” and biologically active (Durber et al. 1976). However, more recent findings show that cortisol dynamics are altered with OC use compared to nonusers (Høgsted et al. 2021). These authors found that the hypothalamus‐pituitary‐adrenal axis response is blunted for the awakening cortisol over 60 min but with a significantly higher absolute cortisol value at awakening with OC use. However, neither low or elevated levels of cortisol appear to influence exercise performance per se (Del Corral et al. 1998; Robertson et al. 2016). We can only surmise that the level of cortisol response in the present study had no appreciable effect on exercise performance at any stage or over the course of the menstrual cycle.

### 4.4 Neuromuscular Responses

The MVC, VA%, and contractile properties were all maintained compared to preexercise measures for the biceps postexercise. This was to be expected as the forearm flexors were relatively inactive during cycling exercise. Conversely, the knee extensor MVC was significantly decreased following cycling exercise in BASE and WARM but not COOL; however, VA% and muscle activity during cycling was maintained across all conditions. The maintenance of VA% agrees with previous studies, although a definitive reason for this seems elusive. Others (De Jonge et al. 2001) examined the female maximal isometric quadriceps strength with superimposed electrical stimulation, isokinetic knee flexion, and extension and hand grip strength in three phases of the menstrual cycle and found that natural fluctuations in estrogen and progesterone did not affect strength parameters, contractile properties, stimulated fatigue, or isokinetic fatigue. Additionally, others (De Souza et al. 1990) have reported no change for muscle strength, fatigue, twitch, and tetanus characteristics throughout the menstrual cycle. Both studies concluded that hormonal fluctuations do not exert an effect on muscle strength or contractile properties; however, the participants were eumenorrheic and had not taken any form of contraceptive or hormone therapy in the 6 months preceding the study.

Although oral contraceptives alter endogenous hormone production, the stable exogenous hormone profile may blunt both beneficial and detrimental effects of fluctuating estrogen and progesterone on neuromuscular function. Given this, we did not anticipate large changes in voluntary activation or contractile properties across OC phases. The maintenance of VA% aligns with this expectation and previous literature (De Jonge et al. 2001). The observed reductions in TPf and contraction duration, markers of peripheral fatigue, likely reflect postexercise alterations in excitation–contraction coupling rather than hormonal or central drive differences, supporting the view that OCs have minimal acute influence on neuromuscular fatigue mechanisms under these conditions.

The contraction duration for knee extensors decreased postexercise in all conditions compared with preexercise values, whereas TPf decreased postexercise in BASE and WARM only. These data suggest the occurrence of peripheral fatigue rather than central fatigue following exercise in different ambient environments at different points during the OC cycle. Peripheral fatigue is characterized by changes at, or distal to, the neuromuscular junction (Enoka and Stuart 1992). This decline in the force‐generating capacity of the skeletal muscle could be due to changes in the cross‐bridge cycling activity, excitation–contraction coupling failure, or failure of action potential propagation, despite unchanged or increasing neural drive (St Clair Gibson et al. 2001; Taylor et al. 1997).

A theory regarding the decline in TPf slowing relates to slower muscle contraction, which is largely dependent on the speed of cross‐bridge interactions (Cannon et al. 2008; Wada et al. 2013; Klitgaard et al. 1989). A decline in contraction duration and TPf was present in all conditions, which suggests that for this parameter, peripheral fatigue is not related to ambient temperature differences as WARM attained a *T*_c_ of ∼38.5°C compared with 38.2°C and 38.3°C for BASE and COOL, respectively.

### 4.5 Limitations

A potential weakness in our experimental design is that we do not have a direct comparison of the performance at 18 days in cooler (20°C) conditions. However, performance was not changed when the women were in the late active pill phase (day 18) with potentially higher endogenous and exogenous estradiol, indicating that performance improvement in cooler temperatures would be unlikely since performance was the same on days 1 and 18 in 20°C. That is, when hormone levels were either low (day 1) or rising (day 8) which would have less impact on body temperature response. Conversely, there would be no expectation for improved performance if ambient temperature was 20°C on day 18 with higher values of endogenous and exogenous estradiol.

Our results should be interpreted with the limitation of not having available the hormone status during the different phases of the menstrual cycle. However, previous studies reporting hormone profiles throughout the pill cycle are highly comparable and provide a reasonable basis for interpretation of our data (Rechichi et al. 2009; Rodriguez et al. 2024; Elliott‐Sale et al. 2021). For example, over the course of the menstrual phase when taking OC, higher levels of exogenous estradiol were observed at 20–21 days of active pill ingestion, but endogenous estradiol decreased during active pill consumption with both exogenous and endogenous progesterone levels remaining stable (Rodriguez et al. 2024). Notably, both endogenous and exogenous estradiol and progesterone at 1, 8, and 18 days were highly stable which correspond to the days when we assessed exercise performance and the neuromuscular properties of our participants. Therefore, we can only conclude that during the testing period and days in our study the behavior of the hormones which might affect thermoregulation and neuromuscular performance were as predicted. Consequently, in the same study, during the 7 days of inactive pill ingestion (21–28 days), the endogenous estradiol level had a sharp rise reaching its highest value on day 28 when exogenous estradiol declined; notably, both exogenous and endogenous progesterone were not markedly different over this same time (see Figures 2 and 3 in (Rodriguez et al. 2024)). Although these authors suggest that their findings challenge the assumption that endogenous and exogenous hormones are stable throughout the 28‐day pill cycle, it appears that the greatest changes occur during the last 8 days of the menstrual cycle.

## 5 Conclusions

In conclusion, our findings suggest that OC use in normal women do not affect the ability to self‐regulate endurance exercise in either warm or moderate ambient conditions, even though *T*_c_ was higher by the end of exercise in the warmer compared with cool conditions. Importantly, we suggest that our study is the first to show that OC use does not detrimentally alter neuromuscular performance in these women across ambient conditions and across days 1–18 of the OC cycle.

## Funding

The authors have nothing to report.

## Conflicts of Interest

The authors declare no conflicts of interest.
